# Losing sleep over work scheduling? The relationship between work schedules and sleep quality for service sector workers^☆^

**DOI:** 10.1016/j.ssmph.2020.100681

**Published:** 2020-10-21

**Authors:** Kristen Harknett, Daniel Schneider, Rebecca Wolfe

**Affiliations:** a UC San Francisco, USA; b Harvard University, USA

**Keywords:** Sleep, Sleep quality, Work scheduling, Unpredictable schedules, United States

## Abstract

In the retail and food service sectors, work schedules change from day-to-day and week-to-week, often with little advance notice, posing a potential impediment to healthy sleep patterns. In this article, we use data from the Shift Project collected in 2018 and 2019 for a sample of over 16,000 hourly workers employed in the service sector to examine relationships between unstable and unpredictable work schedules and sleep quality. We extend prior research on shift work and sleep disruption, which has often focused on the health care sector, to the retail and food service sector, which comprises nearly 20 percent of jobs in the U.S. We find that the unstable and unpredictable schedules that are typical in the service sector are associated with poor sleep quality, difficulty falling asleep, waking during sleep, and waking up feeling tired. As a benchmark, we compare unstable and unpredictable work schedules with two well-known predictors of sleep quality – having a young child and working the night shift. The strength of the associations between most types of unstable and unpredictable work schedules and sleep quality are stronger than those of having a pre-school aged child or working a regular night shift. Chronic uncertainty about the timing of work shifts appears to have a pernicious influence on sleep quality, and, given its prevalence for low-wage workers, potentially contributes to stark health inequalities by socioeconomic status.

## Highlights

• Work schedules in the service sector are often unstable and unpredictable.
• Data from The Shift Project (n=16,000) reveal strong associations between precarious work schedules and sleep quality.
• Unstable work schedules are more predictive of sleep quality than working the night shift or parenting a young child.
• Chronic uncertainty about the timing of work shifts impedes healthy sleep patterns.

Working conditions have important immediate and durable effects on health (Burgard and Lin, 2013), and, for many workers, job quality has significantly deteriorated over the past fifty years (Kalleberg, 2009). The minimum wage has failed to keep pace with inflation and, as a consequence, real wages have eroded markedly (Mishel et al., 2012). The availability and generosity of fringe benefits offered to workers, such as employer-sponsored health insurance and retirement benefits, have also eroded (Hacker, 2006; Kalleberg, 2011). Alongside worsening economic job conditions, many workers also experience considerable irregularity, instability, and unpredictability in their work schedules. The temporal dimensions of work conditions – how many hours, the timing of shifts, and who decides – represent potentially important pathways through which work affects health. In this article, we aim to elucidate channels through which job conditions influence health by focusing on the connections between routine work schedule instability and sleep quality, because sleep is an important determinant of mental and physical health and mortality (Colten et al., 2006; Duncan et al., 2019).

Work schedule instability is prevalent in the U.S. labor force and particularly so in the retail and food service sector, which comprises nearly 20 percent of all jobs in the U.S. economy (Current Population Survey, 2018; Lambert, 2008; Lambert et al., 2014; Carré and Tilly, 2017). Many workers in the service sector are scheduled to work shifts that vary day-to-day and week-to-week with little input from the workers themselves. Temporal precarity – or precariousness in the amount, timing, regularity, and predictability of work time – has received relatively less attention than wages or benefits, but recent emerging evidence suggests that the time dimension of work has widespread consequences for workers’ health and wellbeing (Schneider and Harknett 2019a; Williams et al., 2019). These precarious schedule conditions have been the subject of increased attention and legislative action over the past five years, with some cities and states passing regulations that aim to increase schedule predictability and stability (Wolfe and Cooper, 2018). This policy action has been spurred by an awareness that routine uncertainty in schedules takes a toll on workers.

Routine uncertainty in work schedules is a potential impediment to healthy sleep patterns, because it disrupts schedules and routines, increases worker stress, and leads to work-family conflict (Buxton et al., 2016; Crain et al., 2014; Nicol and Botterill, 2004). Yet, evidence on the connection between precarious work schedules and sleep outcomes is scant because of a lack of available data. Precarious schedule conditions such as short advance notice and last-minute schedule changes have typically not been captured in large-scale surveys and are not available in surveys that include sleep outcomes.

This article harnesses survey data collected by the Shift Project in 2018 and 2019 designed to address this gap. The Shift Project data contain both richly detailed measures of precarious work schedules and several dimensions of sleep quality – self-rated quality, difficulty falling asleep, sleep disturbances, and waking up feeling tired. We estimate associations between each of five dimensions of precarious work schedules and each of four sleep outcomes, and benchmark these associations against two known correlates of poor sleep – parenting a young child and working a regular night shift.

## Background and prior research

### Sleep as a determinant of health and health inequalities

Healthy sleep is increasingly recognized as a “critical pillar of health” (Duncan et al., 2019) and poor sleep quality has been recognized as a serious public health issue (Colten et al., 2006). Those whose sleep duration is too long or too short are at higher risk for chronic disease, mental health problems, accidents, and mortality (Cappuccio et al., 2010; Gallicchio and Kalesan, 2009; Krueger and Friedman, 2009). Insufficient sleep is associated with higher rates of all-cause mortality, heart disease, diabetes, cancer, and other health issues (Cappuchio et al., 2011; Cappuchio et al., 2010; Lieu et al., 2012). Poor sleep quality, such as difficulty falling asleep, staying asleep, or nonrestorative sleep, are associated with increased risk for cardiovascular issues, diabetes, and metabolic syndromes, as well as mental health issues such as the onset of depression and anxiety (Chokesuwattanaskul et al., 2018; Hertenstein et al., 2019; Lian et al., 2019). Sleep insufficiency and poor sleep quality are also associated with cognitive impairment and with a weakened immune response (Cohen et al., 2009; Walker, 2008).

Healthy sleep is unequally distributed across social groups and is strongly associated with poverty and race (Patel et al., 2010). The prevalence of insufficient sleep is higher among people of color, and this disparity has increased over time (Stamatakis et al., 2007). Insufficient sleep and sleep complaints – such as difficulty falling asleep and staying asleep – are associated with lower income and lower levels of educational attainment (Grandner et al., 2010; Krueger and Friedman, 2009). The job conditions experienced by those with lower socioeconomic status could play a role in explaining this strong connection between socioeconomic status and sleep.

### Work as a determinant of health and health inequalities

Given the widespread health consequences of sleep quality and sufficiency, social structural conditions that impede healthy sleep patterns are likely to be important determinants of health and to contribute to health disparities (Hale, 2005; Hale et al., 2015; Knutson, 2013; Laposky et al., 2016). Work is prominent among these social structural conditions that influence sleep and, in turn, health outcomes.

Over the past fifty years, the nature of work has changed dramatically, and job conditions in many sectors have shifted towards lower wages, fewer benefits, and greater job insecurity (Hacker, 2006; Kalleberg, 2009). These changes, collectively, have been characterized as a rise in “precarious” employment - work that is more uncertain and unpredictable for workers (Kalleberg, 2009, p. 2). Although the rise in precarity has been widespread, workers with lower levels of educational attainment have been particularly hard hit by declining wages and benefits, and increased job insecurity (Fligstein and Shin, 2004). While the economic dimensions of precarious work have been treated as paramount (Osterman and Shulman, 2011, p. 4), a set of accompanying changes in the nature of work time have received relatively less attention, yet are also of potential consequence for worker health and wellbeing (Snyder, 2016).

In the modern-day service sector, employers have come to rely on a set of “just-in-time” scheduling practices that lead to a great deal of routine uncertainty for workers (Clawson and Gerstel, 2015). Many workers receive their work schedules with only a few days of notice, and their scheduled shifts are subject to change at the last minute as employers adjust staffing to closely align with consumer demand (Appelbaum et al., 2003; Golden, 2001; Halpin, 2015). Workers also have little opportunity for input into the amount or timing of their work hours, and often work fewer hours than needed to make ends meet (Houseman, 2001; Lambert, 2008). These practices afford employers a great deal of flexibility and the ability to minimize labor costs but lead to chronic uncertainty and instability for employees related to when and how much they will work (Halpin, 2015; Henly Shaefer, and Waxman, 2006). These practices are particularly prominent in the service sector but affect workers in a wide range of industries including health care, hospitality, and logistics (Lambert et al., 2014).

The rise in just-in-time and precarious work schedules has potentially pernicious consequences for the health and wellbeing of workers as well as their families and communities (Benach & Muntaner, 2011). Marmot and Wilkinson in their 2005 edited volume, *Social Determinants of Health,* assert that the effects of unemployment on health are well documented, but the effects of emerging job conditions related to precarious work are not well understood. In their review article, Benach et al. (2014) describe a rich literature on restructuring and downsizing and health, but a less-developed evidence-base on multiple dimensions of employment precarity and health. They call for further articulation of these dimensions and their consequences, a gap that we begin to fill with this paper.

## Mechanisms linking precarious work and sleep quality

Precarious work schedules may affect sleep quality through a variety of pathways – by disrupting routines, causing high strain for workers, and creating work-family conflict. Although we are not able to test the relative importance of this set of mechanisms in our analysis, we describe these mechanisms to demonstrate a set of plausible pathways through which precarious work schedules and sleep quality may be causally related.

*Disrupted Routines*. One of shift work's main impacts on the lives of workers is the disruption of daily routines (Berkman et al., 2014). Regular routines are nearly impossible to maintain when work schedules change from day to day and week to week with little advance notice and little or no input from the worker, as is typical in service sector work (Lambert, 2008). This irregularity in schedules has consequences for sleep given that regularity in daily routines facilitates healthy sleep patterns, and irregularity is associated with insomnia (Moss et al., 2015).

Regularity of lifestyle through regular schedules and routines is connected to the biological circadian rhythm that regulates sleep (Monk et al., 1994). Lifestyle regularity, in turn, is significantly associated with subjective sleep quality across the lifecourse, with prior research documenting this association in an adult sample (Monk et al., 2003), among an elderly sample (Zisberg et al., 2010), and among college students (Carney et al., 2006).

Research shows that shift work has a marked impact on sleep, because it often involves acute sleep loss in connection with night shifts and early morning shifts that disturb circadian rhythm (Kecklund and Axelsson, 2016; Åkerstedt, 2003). Shift workers with unstable schedules cannot ever fully transition their sleep-related circadian rhythms as their schedule changes too often for their bodies to adjust, and as a result, they experience additional health risks (Geiger-Brown et al., 2012). This relationship is evident, for instance, among nurses whose irregular shifts were associated with poor sleep (Neidhammer, Lert, and Marne, 1994). In an in-depth interview study, Coveney (2013) characterizes a relationship between “shift work and broken sleep.” Shift workers in this study report feeling tired all the time but also experiencing difficulty sleeping because their body's clock is out of sync with their work schedule. The sleep challenges that shift workers with irregular or nonstandard schedules face may be exacerbated because sleeping during the day raises their exposure to ambient noise and environmental disturbances (Muzet, 2007).

Overall, shift workers are at a heightened risk of developing chronic sleep disturbances such as insomnia (Moss et al., 2015; Åkerstedt, 2003). Medical professionals have coined the term “shift work disorder” to describe shift workers with the greatest sleepiness and performance impairment at night and insomnia during the day (Åkerstedt and Wright, 2009).

*Job Strain*. Karasek's classic job strain model provides a theoretical framework for understanding how the joint effects of job demands and job control, that is, external requirements and decision-making discretion, affect worker strain (Karasek, 1979). In this model, job strain results from the intersection of high demands and low levels of control and autonomy. Jobs with unstable and unpredictable schedules and with little to no worker control or input into the amount or timing of work hours fall into a “high strain” category, which is predicted to have a host of negative consequences for worker health (Berkman, Kawachi, and Theorell, 2014).

One negative consequence of high job strain is adverse impacts on worker sleep, documented in a wide range of settings. In a prospective study of Swedish workers, Linton (2004) found that job strain doubled the risk of developing sleep difficulties over a year-long follow-up period. Similarly, in a large-scale study of employed men and women in the greater Stockholm area, high job strain was strongly associated with disturbed sleep and not feeling rested upon waking. In the Netherlands, Delange et al. (2009) used longitudinal data to examine this relationship, finding that high strain jobs were associated with sleep difficulties. For these workers, moving from a low to high strain job was associated with an increase in sleep complaints (Delange et al., 2009). Similar results were found in a study of nurses' aides for whom strain (high demands and low control) were associated with poor sleep quality (Eriksen et al., 2008). The literature offers evidence for similar links in the United States. Using panel data from Americans’ Changing Lives Survey, Burgard, and Alishare (2009) find job stress to be associated with poor sleep quality. Adding some nuance to this picture, Magnusson et al. (2011) use structural equation models to examine the relationship between job strain and sleep, and find evidence for reciprocal relationships (Magnuson et al., 2011).

*Work-Family Conflict.* Building on and extending Karasek's job demands/control model, a next generation model of “Work-Family Strain” incorporates conflicting demands from both work and family (Berkman et al., 2014; Bianchi and Milkie 2010; Moen et al., 2015). This model suggests that workers with high work and family demands will encounter strain in meeting all demands, and that this strain will be exacerbated in the context of limited job control. This work-family strain, in turn, has implications for workers' health.

In a sample of IT professionals, employees who experience more work-family conflict report less sleep sufficiency, poorer sleep quality, and more insomnia symptoms (Buxton et al., 2016; Crain et al., 2014). Higher work-family conflict predicted shorter nighttime sleep duration, greater likelihood of napping, and longer nap duration, as well as greater inconsistency of nighttime sleep duration and sleep clock times. Further strengthening the evidence base for the relationship between work-family conflict and sleep, a randomized intervention for IT workers that improved work-schedule flexibility and reduced work-family conflict was also found to improve sleep duration (Olson et al., 2015).

Although the evidence linking work-family conflict and sleep quality for IT professionals is compelling, we lack parallel evidence for other industries including the service sector. We do, however, have evidence that unpredictable and unstable schedules in the service sector lead to work-family conflict. This relationship is documented in a study of 21 retail apparel stores (Henly and Lambert, 2014) and using data from the General Social Survey (Golden, 2015). These studies find that nonstandard hours, irregular work shifts, short advance notice, and on-call schedules are strong predictors of work-family conflict.

## Precarious work schedules and sleep

Prior empirical research on work schedules and sleep have most often focused on schedule flexibility and control and work-family conflict (Kelly and Moen, 2020) or on non-standard night shifts (Presser, 2003). However, the reality of work for millions of Americans, especially those in the retail and food service sectors, is of work schedules that vary from day-to-day and week-to-week, often with little advance notice (Lambert et al., 2014; Schneider and Harknett, 2019a). Employers in these sectors use a set of “just-in-time” scheduling practices to align staffing as closely as possible with customer demand and workers are subject to on-call shifts, last minute changes to shift timing, back-to-back closing then opening (clopening) shifts, and short advance notice of assigned work schedules, all in the context of limited employee schedule control (Lambert, 2008). The existing literature on the association between these aspects of work schedules and sleep quality is much more limited.

On-call schedules (where workers are not present at the job site, but are required by their employer to be available for work and generally only paid if called in) appear to negatively affect worker sleep and life. Employees who work on-call limit their activities, experience stress as their homelife is interrupted, and experience greater psychological disruption and less psychological equilibrium (Nicol and Botterill, 2004). Pilcher and Coplen (2000) found that those working on-call had greater difficulty falling asleep and staying asleep while on-call versus when they were not on-call. Rotating shifts also appear to negatively impact length of sleep, as does having short rest periods between shifts (with curtailment of sleep beginning at 11 h off between shifts) (Åkerstedt, 2003).

Further evidence of the connection between work schedules and sleep comes from a randomized intervention study at The Gap clothing stores. The Gap Study randomly assigned some stores to an intervention that offered employees two-weeks’ notice of schedule, eliminated on-call shifts, allowed tech-enabled shift swapping, and improved consistency in start and end times for employees (Williams et al., 2018). Employees saw greater consistency, predictability, and employee input in their schedules. Prior to the intervention, employees slept an average of 6.2 h on nights they worked and 47% of workers reported that their work schedule interfered with their sleep. Stable scheduling interventions improved sleep by 6–8% on average (Williams et al., 2019). These results also align with Schneider and Harknett's (2019a) findings that retail and food service workers exposed to unstable and unpredictable schedules, including short advance notice and on-call shifts, report lower overall sleep quality.

## Hypotheses

The prior research documents several types of work schedules that are impediments to healthy and sufficient sleep patterns, including inflexible scheduling and shift work. A more limited body of prior research connects sleep quality to a more specific set of “just in time” work scheduling practices that appear widespread in the contemporary service sector.

We build on this prior research and examine a broader set of measures of work schedules than has been used in the literature and we examine the association between such work scheduling exposures and a more comprehensive set of sleep quality outcomes than has been used in the small literature to date on “just in time" scheduling (i.e. Williams et al., 2019; Schneider and Harknett, 2019a).

We hypothesize that on-call work, shift-timing changes, and working back-to-back closing then opening shifts will be associated with worse sleep quality. We also hypothesize that more advance notice of work schedules and more schedule control on the part of workers will be positively associated with sleep quality.

We benchmark the size of these associations against two well-known correlates of sleep disruption: parenting young children (Krueger and Friedman, 2009; Maume et al., 2009; Venn et al., 2008) and working a night shift (Moreno et al., 2019; Åkerstedt and Wright, 2009). A priori, we do not have a prediction about the relative magnitude of these associations. Rather, the benchmarking exercise is designed to give a basis for interpreting the magnitude of the associations between work schedule unpredictability and instability and sleep relative to two known and salient sleep impediments.

## Data and methods

The data for this article comes from the Shift Project survey. The Shift Project has collected eight waves of survey data from workers employed by large retail and food service firms starting in Fall 2016. This article draws on the survey waves collected between Fall of 2018 and Fall of 2019, the waves when the most detailed information on sleep quality was collected. Survey respondents were employed by one of 144 large retail or food service employers and reported that they were paid by the hour.

The Shift Project data collection recruits survey respondents from a set of large, named retail and food service firms. The recruitment approach is to deliver targeted advertisements to employees of large retail and food service firms on Facebook and Instagram. The advertisements are targeted to adults 18 years of age or older, residing in the U.S., who are employed at one of 144 large, named retail or food service employers. The set of 144 retail and food service employers are chosen because they are the top retailers or casual dining establishments by revenue. These firms employ over 50% of the service sector workforce (author tabulations from the Reference USA database).

The use of Facebook as a sampling frame is nontraditional, but the coverage of the Facebook sampling frame compares favorably to traditional approaches such as random digit dialing or address-based sampling (Couper, 2017; Link et al., 2008). Recent estimates show that more than 80 percent of working aged adults are active on Facebook, and that use is not particularly stratified by class, race, or other demographics (Greenwood et al., 2016).

The survey recruitment advertisements name the employer and include the text, “Working at [company name]? Take a Survey and Tell Us about Your Job!” The advertisement displays a picture of a worker in a workplace setting, which is designed to resemble the appearance of the actual worker and setting of the targeted employee. Those who click on the ad are taken to a Qualtrics online survey, which takes between 15 and 20 min to complete. Those who complete the survey have the option of providing their contact information to be entered into a lottery for an iPad.

To address concerns about potential selectivity of survey respondents, the Shift Project study used several strategies, summarized in Schneider and Harknett (2019b). First, to address bias on observed characteristics such as sex, age, race, education, and parental status, post-stratification weights were constructed to align sample demographics of the Shift Project sample with those of service sector workers in the American Community Survey. When these weights were applied, the magnitude and significance of the estimated relationships between schedule attributes and outcomes including sleep quality were largely unchanged (Schneider and Harknett, 2019a). Second, to address bias on unobservables, the Shift Project recruited survey respondents through pairs of opposing advertising message that were designed to select on unobservables, such as overwork versus under-work, loving versus hating one's job, and having good versus bad relationships with managers. Comparisons of samples recruited through separate channels showed that selectivity did not bias the relationships of interest between schedule instability and worker wellbeing outcomes, including sleep quality (Schneider and Harknett, 2019a). Third, estimates from the Shift Project sample were compared to the NLSY97 and CPS and found to be more similar to each of these probability samples than they were to each other (Schneider and Harknett, 2019b).

Given that the survey was self-administered on-line, not all who began the survey completed it. In the analysis in this article, we analyze data for respondents with complete data for all analytic variables in the model (which ranged between 15,075 and 16,316, depending on the dependent variable and set of model covariates). This constitutes 40% of the sample of about 38,500 who began the survey. We compared the characteristics of those who broke off from the survey with those who completed the survey and find that these groups do not vary significantly in any of their reported work characteristics. In other published research using the Shift Project data, results have been consistent whether listwise deletion or multiple imputation approaches were used (Schneider and Harknett, 2019a).

### Dependent variables

We use four separate self-reported measures of sleep quality. First, to measure perceived sleep quality, workers were asked: “During the past month, how would you rate your sleep quality overall?” and were given a choice of four response categories: poor, fair, good, very good. Sleep quality was then coded on a scale ranging from 1 = poor to 4 = very good. For this measure, higher values represent better quality sleep.

Then, workers were also asked three specific questions about sleep difficulty or disturbance. Difficulty falling asleep was captured by a question that asked: “During the past month, how often did you have difficulty falling asleep?” with response categories of never, 1–2 times per month, weekly, multiple times per week, and every day. The difficulty falling asleep scale takes on values from 1 = never to 5 = every day. Workers were asked about sleep disturbances with a question that asked: “During the past month, how often did you wake up repeatedly during sleep?” The sleep disturbance scale ranged from 1 = never to 5 = every day.

Finally, workers were asked about waking up feeling tired: “During the past month, how often did you wake up feeling exhausted/fatigued?” The waking up tired scale also ranged from 1 = never to 5 = every day.

### Independent variables

We use 5 measures of schedule instability and unpredictability:

Workers were asked if they worked an on-call shift in the past month: “In the past month or so, have you ever been asked to be “on-call” for work at [EMPLOYER NAME]? By “on-call”, we mean you have to be available to work, and you find out if you are needed to work just a few hours before your shift.” On-call work is then coded as 1 if yes and 0 if no.

Workers were asked if they ever experienced a change in the timing of their shift in the past month: “In the past month or so, did your employer ever change the timing or the length of your scheduled shift at [EMPLOYER NAME]? For example, your employer asked you to come in early or late, or asked you to leave early or to stay later than the hours you were originally scheduled for.” The indicator of shift-timing changes is coded 1 if yes and 0 if no.

A clopening shift refers to a back-to-back closing then opening shift without at least 11 h in between: “In the past month or so, have you ever worked a closing shift and then worked the very next opening shift with less than 11 h off in between your shifts at [EMPLOYER NAME]? This is sometimes called “clopening.”” Clopening shifts are coded 1 if yes and 0 if no.

We ask respondents the amount of advance notice they are given of their work schedule: “How far in advance do you usually know what days and hours you will need to work at [EMPLOYER NAME]?” We coded their responses into categories of “less than 1 week,” “1–2 weeks,” “2–3 weeks,” “3–4 weeks,” and “4 or more weeks.”

We gauge the extent to which workers have control over their scheduled work hours with a question that asks, “Which of the following statements best describes how the times you start and finish work are decided at [EMPLOYER NAME]?” Schedule control is coded as “no control” if the respondent indicated that they had no input into their start and end times, as “a little control” when employer determined start and end time but the worker could provide some input, as “some control” when worker could set their start and end time within limits, and “a lot of control” when respondents indicated they were free to determine their start and end times for work.

### Control variables

We control for a set of socioeconomic and demographic characteristics including respondent age; male, female, or nonbinary gender identification; educational attainment; school enrollment; household income bracket; whether respondent is married or living with a partner; whether respondent has pre-school-aged children; whether respondent has school-aged or older children; and respondents’ race/ethnicity. By including these covariates, the estimated associations between work schedules and sleep outcomes are net of demographics and socioeconomic characteristics.

We also control for a set of work characteristics that may confound the associations between work schedules and sleep outcomes. Longer tenure on the job may lead to better schedule outcomes but may also have an independent influence on sleep outcomes. Therefore, we control for job tenure at the current job. A broader measure of years of work experience is not available in the Shift Project survey. Hourly wages may be correlated with both work schedules and with sleep outcomes, and we control for workers’ reported hourly wages. We also control for a measure of usual weekly work hours, and whether the respondent reported managerial responsibilities.

Lastly we include fixed effects for survey month to control for seasonality and for respondents’ state of residence to control for geographic variations in work conditions or sleep quality.

### Benchmarking variables

We include parallel analyses of two predictors with which we can benchmark the magnitude of associations between work schedules and sleep outcomes. First, we include having a young child as a predictor of sleep outcomes. This predictor is coded 1 if the respondent reported having a child between the ages of 0 and 4 years of age and 0 otherwise. Second, we include working a regular night shift as a predictor of sleep outcomes. This measure is based on a question that asked respondents to describe their work schedule. Those who reported working a “regular night shift” were coded 1, and all other responses were coded 0.

### Analytic methods

We regress each of the sleep quality dependent variables on an indicator of work schedule instability and control variables using ordinary least squares regression. We use OLS for ease of presentation but have estimated a parallel set of models using ordered logistic regression models, which yield a consistent pattern of results. The OLS results are presented in Table 2, Table 3, Table 4, Table 5.

**Table 1:** Sample descriptives.

	% or mean	SD
Dependent variables		
Sleep quality (1 = poor to 4 = very good)	2.1	(0.89)
Difficulty falling asleep (1 = never to 5 = every day)	3.3	(1.32)
Sleep disturbances (1 = never to 5 = every day)	3.5	(1.30)
Wake up feeling tired (1 = never to 5 = every day)	3.6	(1.27)
Age group		
18–19 years old	14.8	
20–29 years old	35.8	
30–39 years old	14.7	
40–49 years old	11.7	
50–59 years old	15.1	
60–69 years old	6.4	
70+ years old	0.1	
Don't know/refuse	1.5	
Gender		
Male	26.7	
Female	71.7	
Non-binary	1.6	
Race/ethnicity		
Black	4.6	
Hispanic	10.5	
Asian	3.0	
Other	2.4	
Partner status		
Married	25.8	
Lives with partner	19.3	
Not living with partner	54.9	
Has child/ren 0–4 years	9.0	
Has child/ren 5 year or older	31.8	
Educational attainment		
No degree	5.4	
HS degree	33.5	
Some college or more	61.1	
Enrolled in school	28.4	

		% or mean	
Household income group			
Less than $15,000 per year		17.4	
At least $15,000 but less than $25,000		21.2	
At least $25,000 but less than $35,000		17.1	
At least $35,000 but less than $50,000		15.9	
At least $50,000 but less than $75,000		13.4	
At least 75,000 but less than $100,000		7.9	
At least $100,000		7.0	
Years at current job			
less than 1 year		20.3	
1 year		15.3	
2 years		15.4	
3 years		11.4	
4 years		6.8	
5 years		5.7	
6 or more years		25.2	
Usual weekly work hours (number)		31.7	(11.30)
Hourly wage ($)		12.8	(5.01)
Is a manager		21.0	
Works regular day shift		23.3	
Works regular evening shifts		8.3	
Works regular night shifts		8.5	
Works a variable or rotating schedule		60.0	
N		16,316

**Table 2:** Self-rated sleep quality (1 = Poor to 4 = Very good) regressed on work schedule instability.

Works on-call		−0.121	***								
		(7.32)									
Shift-timing change				−0.193	***						
				(12.78)							
Works clopening shift						−0.194	***				
						(13.48)					
Less than 1 week schedule notice (reference)											
1–2 weeks' schedule notice								0.0673	***		
								(3.78)			
2–3 weeks' schedule notice								0.0998	***		
								(5.16)			
3–4 weeks' schedule notice								0.135	***		
								(5.22)			
4+ weeks' schedule notice								0.142	***		
								(4.18)			
No schedule control (reference)											
A little schedule control										0.18	***
										(11.72)	
Some schedule control										0.185	***
										(7.34)	
A lot of schedule control										0.256	***
										(4.61)	
N		16,171		16,316		16,263		16,222		16,025	

T-statistics in parentheses. *p < 0.05, **p < 0.01, ***p < 0.001.

All models control for the socioeconomic, demographic, and job characteristics listed in Table 1.

**Table 3:** Has difficulty falling asleep (1 = Never to 5 = Every day) regressed on work schedule instability.

Works on-call		0.283	***								
		(11.42)									

Shift-timing change				0.309	***						
				(13.65)							
Works clopening shift						0.306	***				
						(14.20)					
Less than 1 week schedule notice (reference)											
1–2 weeks' schedule notice								−0.101	***		
								(3.76)			
2–3 weeks' schedule notice								−0.117	***		
								(4.04)			
3–4 weeks' schedule notice								−0.158	***		
								(4.07)			
4+ weeks' schedule notice								−0.173	***		
								(3.41)			
No schedule control (reference)											
A little schedule control										−0.226	***
										(9.79)	
Some schedule control										−0.246	***
										(6.51)	
A lot of schedule control										−0.438	***
										(5.21)	
N		15,342		15,485		15,430		15,393		15,222	

T-statistics in parentheses. *p < 0.05, **p < 0.01, ***p < 0.001.

All models control for the socioeconomic, demographic, and job characteristics listed in Table 1.

**Table 4:** Sleep disturbance (1 = Never to 5 = Every day) regressed on work schedule instability.

Works on-call	0.199	***								
	(8.07)									
Shift-timing change			0.269	***						
			(11.97)							

Works clopening shift					0.24	***				
					(11.18)					
Less than 1 week schedule notice (reference)										
1–2 weeks' schedule notice							−0.0878	***		
							(3.31)			
2–3 weeks' schedule notice							−0.09	**		
							(3.14)			
3–4 weeks' schedule notice							−0.139	***		
							(3.63)			
4+ weeks' schedule notice							−0.174	***		
							(3.46)			
No schedule control (reference)										
A little schedule control									−0.202	***
									(8.81)	
Some schedule control									−0.211	***
									(5.62)	
A lot of schedule control									−0.261	**
									(3.12)	
N	15,197		15,336		15,282		15,244		15,075	

T-statistics in parentheses. *p < 0.05, **p < 0.01, ***p < 0.001.

All models control for the socioeconomic, demographic, and job characteristics listed in Table 1.

**Table 5:** Wake up feeling tired (1 = Never to 5 = Every day) regressed on work schedule instability.

Works on-call	0.168	***								
	(7.07)									
Shift-timing change			0.291	***						
			(13.50)							
Works clopening shift					0.257	***				
					(12.48)					
Less than 1 week schedule notice (reference)										
1–2 weeks' schedule notice							−0.0833	**		
							(3.27)			
2–3 weeks' schedule notice							−0.137	***		
							(4.98)			
3–4 weeks' schedule notice							−0.181	***		
							(4.92)			
4+ weeks' schedule notice							−0.174	***		
							(3.61)			
No schedule control (reference)										
A little schedule control									−0.246	***
									(11.21)	
Some schedule control									−0.207	***
									(5.77)	
A lot of schedule control									−0.467	***
									(5.87)	
N	15,195		15,335		15,282		15,243		15,079	

T-statistics in parentheses. *p < 0.05, **p < 0.01, ***p < 0.001.

All models control for the socioeconomic, demographic, and job characteristics listed in Table 1.

To convey the strength of the association between each indicator of work schedule instability and each sleep dependent variable, in discussing the results we translate the model coefficients into effect sizes measured in standard deviations (effect size = OLS coefficient/standard deviation of the dependent variable). We also estimate predicted values of the sleep dependent variable in the presence and absence of an indicator of schedule instability. These results are presented in Fig. 2 through 5.

In Table 6, we present the estimated relationships between work schedules and sleep outcomes alongside a parallel set of relationships between parenting a young child or working the night shift and these same sleep outcomes. These comparisons provide a benchmark against which to compare the magnitude of the estimated relationships between work schedules and sleep. We test the statistical significance of the difference between coefficients for scheduling predictors compared with having a young child or working the night shift using Clogg tests (Clogg et al., 1995).

**Table 6:** Marginal effects of work schedule indicators and benchmarking variables on sleep outcomes.

	Sleep Quality (1 = poor to 4 = very good)			Difficulty falling asleep (1 = never to 5 = every day)			Sleep disturbances (1 = never to 5 = every day)			Wake up feeling tired (1 = never to 5 = every day)		
Work Schedule Indicators												
On call schedule	−0.121	***	c	0.283	***	c,n	0.199	***	n	0.168	***	
	(7.32)			(11.42)			(8.07)			(7.07)		
Shift timing changes	−0.193	***	n	0.309	***	c,n	0.269	***	n	0.291	***	n
	(12.78)			(13.65)			(11.97)			(13.50)		
Clopening shift	−0.194	***	n	0.306	***	c,n	0.24	***	n	0.257	***	n
	(13.48)			(14.20)			(11.18)			(12.48)		
Less than 1 weeks' notice	−0.142	***		0.173	***	c	0.174	***	n	0.174	***	
	(4.18)			(3.41)			(3.46)			(3.61)		
No schedule control	−0.256	***	n	0.438	***	c,n	0.261	**	n	0.467	***	c,n
	(4.61)			(5.21)			(3.12)			(5.87)		
Benchmarking Variables												
Has child 0–4 years of age	−0.192	***		0.0442			0.24	***		0.238	***	
	(7.53)			(1.15)			(6.31)			(6.49)		
Works a regular night shift	−0.097	***		0.143	***		0.034			0.106	**	
	(3.89)			(3.84)			(0.91)			(2.98)		

T-statistics in parentheses. *p < 0.05, **p < 0.01, ***p < 0.001.

Note: A Clogg test was used to test the statistical significance of the differences between coefficients for schedule predictors and benchmarking variables. Statistically significant differences are indicated by “c” for having a young child and “n" for working the night shift.

In presenting our results, we use a p-value threshold of p < 0.05 for statistical significance. When we apply a Bonferroni correction (VanderWeele and Mathur, 2019) to account for multiple comparisons, the threshold for statistical significance becomes p < 0.0025 (0.05/20 tests). Even at this lower threshold, nearly all estimated relationships between work schedules and sleep remain statistically significant.

## Results

**Fig. 1:**
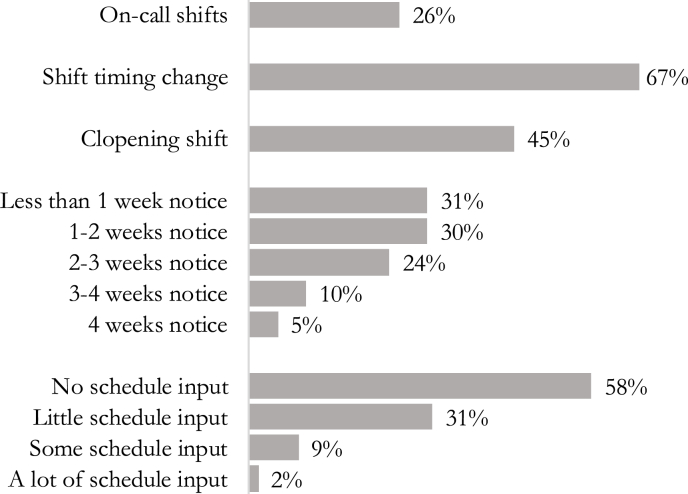
Prevalence of work schedule experiences in shift project survey sample, 2018–2019.

Fig. 1 shows the prevalence of each of the work schedule independent variables. Overall, many workers experience work schedules that are unpredictable and unstable, and most have little if any input into their work schedules. As shown in the figure, 26% of workers reported working on-call shifts, 67% experienced changes in the timing of their work shifts, and 45% worked a back-to-back closing then opening shift. About 60% of workers typically receive less than two weeks of advance notice of their work schedules, and almost 60% report having no input into the times that they start and end work.

Table 1 shows sample descriptives. About half of the sample is between 18 and 29 years of age, another quarter is in their 30s or 40s, and the remaining quarter is aged 50 years or older. The sample is mostly female (72%). More than half the sample has an annual household income less than $35,000, and the average hourly wage is $12.80. About half the sample has held their current job for two years or less.

Table 2 presents the regression estimates summarizing the relationship between work schedules and self-rated sleep quality. Each one of the scheduling independent variables is a significant predictor of sleep quality. Working an on-call schedule is associated with a 1/10 of a point reduction in sleep quality (0.14 standard deviations), and shift-timing changes and working a clopening shift are associated with about a 2/10 of a point reduction (0.22 standard deviations) on a 4-point scale. Having at least 3 weeks' advance notice of one's work schedule (as compared with less than 1 week) is associated with about a 0.14 point increase (0.16 standard deviations) in average sleep quality. Having at least some schedule control is associated with almost 2/10 of a point (0.21 standard deviations) improvement in sleep quality and having a lot of schedule control is associated with almost a 3/10 of a point (0.29 standard deviations) improvement on the 4-point sleep quality scale.

**Fig. 2:**
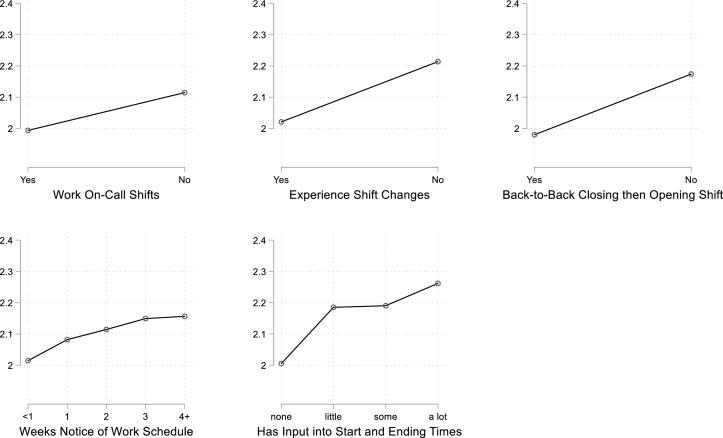
Sleep quality (1 = Poor to 4 = Very good) with and without schedule instability.

Fig. 2 presents these results graphically, showing the model predictions for the average sleep quality score for workers with different work schedule conditions. Workers with unstable and unpredictable work schedules (on-call, shift changes, clopening shifts, and less than 1 weeks' notice) or with no schedule control have “fair” sleep quality on average (a value of 2 on the 4-point scale). Their counterparts with more stable and predictable work schedules (no on-call, no shift changes, no clopenings, and more than 1 weeks’ advance notice) have significantly higher ratings on the sleep quality scale, with scores (2.1–2.3) that are part of the way towards the value of “good” sleep quality (a value of 3).

Table 3 turns to results for reports of difficulty falling asleep. For this outcome, workers reported whether they had difficulty falling asleep on a scale that ranged from 1 = never to 5 = every day. Here we see that workers with on-call schedules, shift-timing changes, or clopening shifts experience more difficulty falling asleep than their counterparts without these types of work schedules. Each of these scheduling conditions is associated with a 3/10 of a point (or around a 0.22 standard deviation) increase on the 5-point difficulty-falling-asleep scale. Having 3 or more weeks of advance notice of one's work schedule or some schedule control are associated with almost a 2/10 of a point decrease (0.12 standard deviations) in difficulty falling asleep. Having a great deal of schedule control is associated with more than 4/10 of a point (or, one-third of a standard deviation) decrease.

**Fig. 3:**
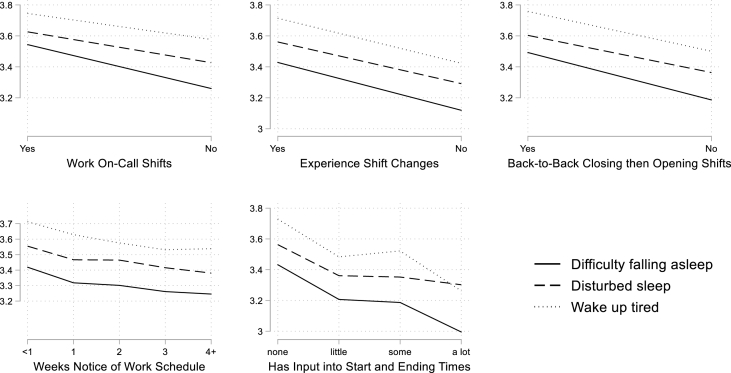
Frequency of Sleep Problems (1 = Never to 5 = Every day) with and without Schedule Instability.

Fig. 3 displays these results graphically. Here we see that those with unstable or unpredictable work schedules or no schedule input report an average of 3.4–3.5 on the 5-point scale for difficulty falling asleep. That score suggests that these workers have difficulty falling asleep more than once a week. For workers with more stable and predictable schedules, the score on the scale is closer to the value for once a week.

Table 4 and Fig. 3 present a parallel set of results for experiencing sleep disturbances, or periods of waking up during sleep. The sleep disturbance scale also ranges from 1 = never to 5 = every day. The pattern of results are similar to those for difficulty falling asleep. Each indicator of work schedule instability, unpredictability, or lack of schedule control is associated with more wakefulness during sleep. The associations are statistically significant and typically around 2/10 to 3/10 of a point in magnitude on the 5-point scale. In standard deviation terms, these relationships range between 0.11 and 0.21 of a standard deviation in magnitude.

Table 5 and Fig. 3 present results for reports of waking up feeling tired. As with previous outcomes, each of the work schedule conditions is significantly associated with reports of waking up feeling tired. The associations ranged from about 2/10 to 3/10 of a point on the 5-point scale (or between 0.13 and 0.23 standard deviations) for on-call work, shift-timing changes, and working a clopening shift, and for having 3 weeks' notice of one's work schedule. Having a lot of schedule control was associated with 5/10 of a point reduction (0.37 standard deviations) in waking up feeling tired.

**Fig. 4:**
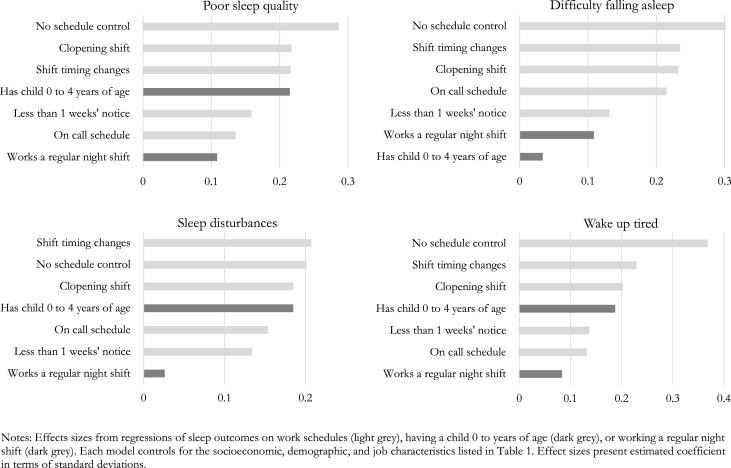
Effect Sizes for Relationships between Schedules, Children, or Night Shifts and Sleep Outcomes Notes : Effects sizes from regressions of sleep outcomes on work schedules (blue bars), having a child 0 to years of age (orange), or working a regular night shift (green). Each model controls for the socioeconomic, demographic, and job characteristics listed in Table 1. Effect sizes present estimated coefficient in terms of standard deviations. . (For interpretation of the references to color in this figure legend, the reader is referred to the Web version of this article.)

The final set of results in Table 6 provide a benchmark against which to compare the associations between work schedule conditions and sleep outcomes. In this table, we present the associations between precarious work schedule and sleep outcomes compared with the associations between having a child between the age of 0 and 4 years or working the night shift and the same sleep outcomes. These relationships are also displayed graphically in Fig. 4.

The associations between parenting young children and worse sleep quality are well documented in the literature (Hagen et al., 2013; Richter et al., 2019). We find that parents of young children rate their sleep quality lower, report more frequent sleep disturbances, and report more frequently waking up feeling tired. Having a young child is not associated with difficulty falling asleep. Table 6 allows us to compare the magnitude of these associations with those of work schedule conditions and sleep outcomes. Among the 20 estimated associations between work schedules and sleep outcomes, 14 of 20 are as large or larger in magnitude than the association between parenting a young child and the corresponding sleep outcome. For 7 of the estimated associations between work schedules and sleep, the magnitude of relationships were statistically significantly larger compared with having a young child. The associations between on-call schedules or less than 1 weeks’ notice and sleep outcomes were smaller in magnitude compared with parenting a young child. However, last-minute changes to shift timing, clopening shifts, and lacking schedule control were each more strongly related to sleep outcomes compared with parenting a young child.

Finally, we compared the associations between working a regular night shift and sleep outcomes. Here, we found an even more striking pattern of results. The set of prevalent scheduling conditions in the service sector – on-call shifts, last minute schedule changes, clopenings, short advance notice, and lack of schedule control – are each stronger correlates of poor sleep outcomes than working a regular night shift. For 15 of 20 estimated relationships between schedules and sleep, the differences in coefficients compared with working the night shift were statistically significant.

## Discussion

Job quality for millions of Americans in the retail and food service sector is defined not just by low-wages and few fringe benefits, but also by work schedules that are unstable and unpredictable. The on-call shifts, last minute changes to schedule timing, short advance notice, back-to-back closing then opening (clopening) shifts, and little schedule control that these workers experience may have important consequences for their health and wellbeing. Whereas prior research on work and sleep has often focused on working non-standard shifts at nights or on weekends, we show how a range of emergent scheduling practices, which lead to routine schedule uncertainty, affect sleep quality.

Drawing on data from The Shift Project, we find that workers who are exposed to these “just in time” work scheduling practices experience worse sleep quality overall, more difficulty falling asleep, more sleep disturbances, and are more likely to feel tired when they wake. We did not have a priori expectations about which dimensions of sleep quality would be most strongly related to work schedule conditions. Likewise, a priori, we did not have strong expectations about which detailed schedule conditions would be most strongly related to sleep outcomes. Overall, we find quite consistent patterns across measures: each measure of work schedule precarity was associated with each measure of sleep problems. Comparing the strength of the associations, we find that three dimensions of work schedules are particularly strongly associated with sleep outcomes: lacking control over the timing of work, working back-to-back closing then opening shifts, and experiencing last-minute changes to scheduled work shifts. The finding for schedule control aligns with previous research for different occupational sectors (i.e. Kelly and Moen, 2020; Eriksen et al., 2008). The finding for clopening shifts and timing changes is novel and noteworthy. These two schedule features are normative in the service sector and strongly associated with sleep problems.

Strikingly, these associations are often greater in magnitude than the well-known negative associations between being a parent to a young child or working a night shift and sleep quality. Much of the prior literature on work schedules and sleep quality has focused on the negative effects of night shifts, motivated by the observation that such schedules can interfere with circadian rhythms. Our results here show that work schedule irregularity and unpredictability are even stronger predictors of sleep quality. These results should encourage future researchers to conceptualize “shift work” more broadly than simply “standard” and “non-standard” shifts and to account for exposure to on-call shifts, clopening shifts, last minute changes to schedule timing, and short advance notice.

These results reinforce existing literature that shows that while employer “just in time” scheduling practices may boost profit margins (Lambert, 2008), they exact a real cost for worker health and wellbeing (Schneider and Harknett, 2019a). These consequences of routine uncertainty in work schedules, among a workforce that is disproportionately female and comprised of workers of colorin amo, may depress population health and widen health disparities.

Policymakers have already begun to consider and implement laws to regulate these sorts of unstable and unpredictable work scheduling practices. Laws in San Francisco, Seattle, New York City, Philadelphia, and Chicago require large retail and food service employers to provide at least two weeks’ of advance notice and regulate on-call and clopening shifts. While these laws clearly fall within the domain of labor policy, such legislation also tackles, as we have shown here, important social determinants of health.

Our work is subject to some important limitations. Our approach to data collection provides data on measures that has previously been unavailable. However, our measures are survey-based and lack the precision and frequency of measurement that one might be able to obtain from either administrative records on work schedules or from actigraphic measures of sleep. Another measurement limitation is that we lack information on a full set of job demands and control, which are known to affect worker health (Karasek, 1979). We also rely on measures that are self-reported by respondents and we cannot rule out some amount of single-respondent bias if some are more negative than others, even when objective conditions are similar. Second, our data is drawn from a non-probability sample. While we cannot fully eliminate the risk of bias in the estimates, prior work has subjected the data to a battery of validation and verification checks (Schneider and Harknett, 2019a, 2019b). Third, our sample is limited to employees of large retail and food firms. While this sampling design limits external validity, the covered population is of considerable public and policy interest as it is precisely the employees of these firms who are covered by recently enacted legislation. Finally, we report associations and not causal effects. However, we note that these associations are robust to a large set of controls for observable potential confounders and that the scope of heterogeneity in the sample is limited by design – in that everyone is an hourly worker at the same circumscribed set of large firms -- eliminating many sources of potential confounding.

This article contributes to the literature on work as a social determinant of health. Building on a rich tradition of research on how work at non-standard hours and shift work can interfere with sleep quality, we show that a constellation of work schedule conditions that are prevalent in the large U.S. service sector are each related to unhealthy sleep patterns. Our research suggests that retail and food service employers’ drive to minimize labor costs through scheduling work shifts on short notice, making last-minute adjustments to work schedules, and limiting worker choice about their start and end times is likely to lead to less healthy sleep patterns for the workers subject to these scheduling conditions.

## CRediT authorship contribution statement

**Kristen Harknett:** Conceptualization, Methodology, Formal analysis, Investigation, Writing - original draft, Writing - review & editing, Funding acquisition. **Daniel Schneider:** Conceptualization, Methodology, Investigation, Writing - original draft, Writing - review & editing, Funding acquisition. **Rebecca Wolfe:** Writing - original draft, Writing - review & editing, Visualization.

## Declaration of competing interest

The authors declare that they have no known competing financial interests or personal relationships that could have appeared to influence the work reported in this article.
